# Preventing Breast Cancer-Related Lymphedema in High-Risk Patients: The Impact of a Structured Surveillance Protocol Using Bioimpedance Spectroscopy

**DOI:** 10.3389/fonc.2018.00197

**Published:** 2018-06-12

**Authors:** Pat W. Whitworth, Chirag Shah, Frank Vicini, Andrea Cooper

**Affiliations:** ^1^Nashville Breast Center, Nashville, TN, United States; ^2^Cleveland Clinic, Department of Radiation Oncology, Taussig Cancer Institute, Cleveland, OH, United States; ^3^Michigan Healthcare Professionals, 21st Century Oncology, Farmington Hills, MI, United States

**Keywords:** breast cancer, lymphedema, bioimpedance, surveillance, early detection, axillary lymph node dissection

## Abstract

**Purpose:**

We evaluated the impact of structured surveillance using bioimpedance spectroscopy (BIS) to reduce the rate of chronic breast cancer-related lymphedema (BCRL) in high-risk patients undergoing axillary lymph node dissection (ALND).

**Methods:**

From April 2010 through November 2016, 93 patients who underwent ALND were prospectively monitored with BIS using L-Dex. Intervention for an L-Dex increase of >10 consisted of applying an over the counter (OTC) sleeve followed by re-evaluation after 4 weeks. The utilization of complex decongestive physiotherapy (CDP) represented a surrogate for chronic BCRL.

**Results:**

Median follow-up was 24 months. 55% of patients received taxane-based chemotherapy, 24% received some form of axillary irradiation (includes additional fields or high tangents) and 66% had an elevated body mass index (BMI) with the median number of nodes removed being 19. Overall, 75% of these patients had at least one additional high-risk feature (taxane chemotherapy, axillary radiation, elevated BMI), 48% had at least two, and 6% had all. Thirty-three patients (35.4%) developed an elevated L-Dex score with only 10 (10.8%) requiring CDP (30.3% of those undergoing treatment with sleeve). At last follow-up, only three patients (3%) had unresolved BCRL.

**Conclusion:**

The results of this analysis support previous data regarding prospective BCRL surveillance and early intervention using BIS. With this approach, only 3% of patients have chronic BCRL.

## Introduction

Survival for women with breast cancer has continued to improve significantly over the past few decades (1). As a result, greater emphasis has been directed toward breast cancer survivorship and the management of the long-term sequelae of treatment (2). One of the most feared of these sequelae is breast cancer-related lymphedema (BCRL) of the arm; a condition that can lead to a detriment in quality of life and increased morbidity (3, 4). The risk of developing BCRL varies significantly based upon the receipt of locoregional and/or systemic therapies with increased BCRL rates observed with mastectomy, axillary lymph node dissection (ALND), regional nodal irradiation (RNI), and taxane-based chemotherapy (3–5). Additionally, patient-specific factors including elevated body mass index (BMI) may contribute to the risk of developing BCRL (3, 4). Programs designed to detect and prevent progression of BCRL must identify patients with high-risk features to allow for early, non-invasive treatment approaches, prior to clinical progression of BCRL; while screening all patients is an option, this would require much greater and higher costs while including a group at low risk for developing BCRL (6, 7). By identifying patients at high risk for BCRL, a pre-emptive strategy can employ less intense, more cost-effective therapies when BCRL is diagnosed sub-clinically (6, 7), while reducing the impairment in quality of life associated with progression of BCRL.

Despite the more frequent use of sentinel lymph node biopsy (SLNB) over the past two decades, some patients with more advanced axillary disease still require ALND. These patients are also more likely to receive additional therapies (i.e., RNI, chemotherapy), further increasing their risk of developing chronic BCRL (3, 4). Recent NCCN guidelines for breast cancer survivorship recommend educating, monitoring, and treating breast cancer patients for BCRL (8). Prospective surveillance with bioimpedance spectroscopy (BIS) is one method that has been used successfully to detect subclinical BCRL, permitting simple, early intervention before progression to chronic BCRL (6, 7). Since 2010, we have used BIS to monitor patients for BCRL and intervene at an earlier point with the intent to reduce the rate of chronic BCRL. To explore the potential effect of this structured surveillance program on BCRL, we analyzed the rate of development of BCRL in a group of high-risk patients undergoing ALND.

## Materials and Methods

Between April 2010 and November 2016, 596 patients were enrolled in a prospective BCRL surveillance program using the L-Dex U400 Device (ImpediMed, Brisbane, Australia) at a single institution (Nashville Breast Center, Nashville, TN, USA). Results for all patients have been previously reported (9). In short, inclusion criteria included patients with breast cancer undergoing surgery (either breast conservation or mastectomy). Patients undergoing SLNB and ALND were included in the program; however, for the purpose of this analysis, only patients undergoing ALND were evaluated. Exclusion criteria for the prospective program included bilateral disease, electronic devices (i.e., pacemakers), pregnancy, renal failure, and heart failure. Decisions regarding radiation therapy, chemotherapy and endocrine therapy were made at the treating physicians’ discretion but were documented. Collection of data and the analysis was given institutional review board (IRB) approval [WIRB Exemption Determination under 45 CFR 46.101(b)(4)].

Patients enrolled in the prospective surveillance program underwent a standardized BCRL assessment protocol. Pre-operatively, patients had an L-Dex measurement as well as BCRL education. Subsequently, post-operatively, patients underwent L-Dex measurements at 1.5 weeks and then at 3, 6, 9, and 12 months post-operatively followed by bi-annual measurements. The L-Dex measurement technique was based on previous publications from Vicini et al. (10). At any time point, patients were considered to have an elevated L-Dex score if the score increased by at least 10 points from baseline (10, 11). Patients having an increase of more than 10 units were subsequently instructed to utilize an over the counter (OTC) compression sleeve for 4 weeks followed by re-assessment, consistent with current guidelines and prospective data (6, 11). Patients with persistent elevation in spite of compression garment utilization or those that developed clinical BCRL were considered for complex decongestive physiotherapy (CDP). Patients who underwent BCRL treatment without having an L-Dex increase of greater than 10 were also documented.

This analysis focused on the subset of patients undergoing ALND (*n* = 93). Chronic BCRL was defined as the need for CDP. Additional high-risk factors beyond ALND were assessed including elevated BMI (BMI > 25), axillary radiation, or taxane-based chemotherapy. Descriptive statistics are reported as mean (SD), median, and range. Differences between groups were tested using Wilcoxon rank sum tests for quantitative variables and chi-squared tests for categorical variables. Analyses were performed using R version 3.2 or higher. A *p*-value less than 0.05 was considered statistically significant.

## Results

### All Patients (Overall Experience)

The median age of the overall cohort was 55 years old; with respect to surgery, 59% of patients underwent mastectomy and 81% SLNB. With respect to adjuvant therapy, 43% of patients received chemotherapy (27% taxane) and 34% received whole breast/chest wall radiation therapy (4% high tangent, 3% RNI). When evaluating risk factors, 80% of patients (*n* = 475) had at least one high-risk factor [BMI (*n* = 379), ALND (*n* = 93), RNI (*n* = 17), and taxane chemotherapy (*n* = 163)]. One-third of all patients had more than one high-risk factor. The rate of unresolved BCRL was also higher for those undergoing ALND (11% vs. 1%, *p* < 0.001).

### ALND Patients Only

Patient characteristics for this cohort of 93 patients are presented in Table 1. Median age of the cohort was 53 years old. Eighty five percent of patients underwent mastectomy and the remainder breast conserving therapy. The median number of nodes removed was 19 (range: 5–41) and the median number of positive nodes was 3. With respect to additional risk factors (Table 2), 55% of patients received taxane-based chemotherapy, 24% received some form of axillary RT (15% high tangents and 9% comprehensive RNI) and 66% had an elevated BMI. Overall, 75% of these patients had at least one additional high-risk feature, 48% had at least two, and 6% had 3 (either taxane chemotherapy, axillary RT, or elevated BMI). Median follow-up was 24 months (range: 0.3–206.4 months).

**Table 1:** Characteristics of patients.

	ALND	All Patients
Number	93	596
Age	53 (32–87)	55 (28–90)
Mastectomy	79 (85%)	343 (59%)
**BMI**		
Elevated	61 (66%)	372 (67%)
Median	28	27
**Systemic therapy**		
Adjuvant chemotherapy	49 (53%)	155 (26%)
Neoadjuvant therapy	20 (22%)	101 (17%)
Taxane chemotherapy (any time)	51 (55%)	163 (27%)
Targeted therapy (herceptin/TKI)	10 (11%)	55 (9%)
**Radiation therapy**		
Breast/chest wall irradiation	39 (42%)	203 (34%)
High tangents	14 (15%)	26 (4%)
Regional nodal irradiation	8 (9%)	17 (3%)
APBI	0 (0%)	103 (17%)

*ALND, axillary lymph node dissection; SLNB, sentinel lymph node biopsy; BMI, body mass index; APBI, accelerated partial breast irradiation*.

**Table 2:** Additional high-risk features in ALND cohort.

High-risk feature	% of patients
Taxane chemotherapy	55% (*n* = 51)
Axillary radiation	24% (*n* = 22)
High tangents	15% (*n* = 14)
Regional nodal	9% (*n* = 8)
Elevated BMI (>25)	66% (*n* = 61)

*BMI, body mass index*.

Thirty-three patients (35%) undergoing ALND developed an elevated L-Dex score at some point during follow-up. Ten of these patients (11%) went on to require CDP at any point after treatment. At last follow-up, however, only three patients (3%) have required continued therapy (Table 3). The other seven patients remain without evidence of chronic BCRL (97% of all patients remain without evidence of BCRL).

**Table 3:** Status at last follow-up of patients requiring complex decongestive physiotherapy.

Patient #	Follow-up (mos)^b^ (months)	Status at last follow-up
1	10	Continues with CDP
2	56	Discontinued CDP, breast cancer-related lymphedema (BCRL) stable—no additional progression
3^a^	24	Discontinued CDP, BCRL stable—no additional progression
4	84	Continues with CDP
5	30	Discontinued CDP, BCRL stable—no additional progression
6	20	Discontinued CDP, BCRL stable—no additional progression
7	68	Continues with CDP
8	57	Discontinued CDP, BCRL stable—no additional progression
9	68	Discontinued CDP, BCRL stable—no additional progression
10	12	Discontinued CDP, BCRL stable—no additional progression

*^a^Deceased*.

*^b^Time from diagnosis*.

*CDP, complex decongestive physiotherapy*.

## Discussion

The results of the current analysis add to the growing body of outcomes-based evidence that prospective BCRL surveillance and early intervention using BIS is associated with very low rates of progression to chronic BCRL. Of the 93 high-risk patients treated with ALND and prospectively followed and managed in this structured BCRL protocol, only 10.8% required CDP. More importantly, only three of these patients (3%) went on to require additional therapies and 97% remain without evidence of chronic BCRL. These outcomes are superior to contemporary studies of conventional measures reporting BCRL rates in patients undergoing ALND (Table 4) and support structured BIS surveillance to as a method reduce the rate of chronic BCRL (12–17). Additionally, the chronic BCRL rates seen in the present study are lower than those seen with in studies evaluting rates of BCRL in patients with risk factors included in the analysis; for example, Lee et al. found a 23% rate of BCRL at 6 months with taxane-based therapy (3). Similarly, review of the literature has found BCRL rates of 15–60% following mastectomy and 20–60% following RNI, while Ridner et al found a 3.6× increase in the rates of BCRL at 6 months for those with elevated BMI (4, 18). However, the results of our high-risk population demonstrated lower rates of chronic BCRL using a prospective BCRL surveillance program with L-Dex.

**Table 4:** Published rates of breast cancer-related lymphedema after axillary lymph node dissection (ALND).

	Lymphedema incidence	Method of diagnosis
NSAPB B32 (12)	14% at 3 years	Water displacement
Denmark (13)	16–18% at 3 years	Circumference
University of Sydney (14)	18.2%^a^ at 1.5 years	Bioimpedance spectroscopy (BIS)
University of Pittsburgh (15)	12.2% at 1.8 years	Bioimpedance spectroscopy
Italy (16)	27% at 4.2 years	Circumference
AMAROS (17)	23% at 5 years	Circumference
Current study	12% all/35% ALND at 2 years	Bioimpedance spectroscopy

*^a^>5 nodes removed*.

### BCRL Surveillance

The concept of prospective surveillance is driven by the idea that identifying BCRL at the subclinical phase of the process and initiating early, conservative intervention reduces chronic morbidity and the need for invasive and costly procedures (6, 11, 19). If diagnosis in the subclinical phase of BCRL is needed to allow for early conservative therapy, BCRL diagnostic modalities with high sensitivity (ex. perometry, BIS) are needed as compared to traditional techniques (3, 4, 19–21). In recognition of these findings, evidence-based guidelines have been developed to provide clinicians with trigger points using L-Dex scores in the clinic to initiate simple preemptive management (11). Moving forward, additional data employing this surveillance and intervention BIS protocol (in large groups of patients with high-risk features-such as in this analysis) should help to quantify the magnitude of improvements in the long-term outcomes of such approaches with respect to chronic BCRL, quality of life, toxicity, and cost.

At this time, randomized, prospective, and single-institution data have been published supporting early detection and intervention for BCRL (6, 7, 19, 22, 23). Recently, Soran et al. published results with such an approach using BIS, finding low rates of BCRL (6). One key to such an approach is to target patients at high risk of BCRL so they can be provided with the most effective prospective surveillance, while offering clinicians and clinics less intensive options for lower risk patients (ex. less frequent surveillance, clinical surveillance only). Our results support this approach for high-risk patients as 35% has elevated L-Dex levels and 11% required CDP. Moving forward, such a strategy would allow for the effective and efficient assessment of BCRL, providing a potential for cost savings compared with delayed detection and intervention, which has been associated with high costs (24). As such, use of L-Dex would be considered to have an appropriate cost benefit ratio by offering a low cost BCRL diagnostic technique that reduces the high costs associated with chronic BCRL (11, 24). Studies have defined patient and treatment characteristics associated with a high risk of BCRL; future studies will help to further refine identification of subsets of patients that demonstrate the greatest benefit to a careful preemptive approach.

It is important to note that all patients that required CDP were identified with an elevated L-Dex score with the majority of the patients treated with an OTC compression sleeve for 4 weeks. One observation may be that those that progressed to CDP may not have if preventative intervention had been applied earlier. One strategy to allow for even earlier detection would be to reduce the threshold for intervention. This is supported by growing data that support the use of a 2 SD threshold (L-Dex > 7) to initiate intervention as compared to the traditional 3 SD threshold that has been used in the past (25–27). The emerging available data suggest a higher sensitivity to detect mild to moderate volume changes, would facilitate even earlier BCRL treatment.

### Study Limitations

There are obvious limitations to the present analysis. The data for this study was collected prospectively, but the ALND subgroup analyses were performed retrospectively and as such are limited by the biases of such an approach. Additionally, there were a small number of events, limiting the ability to discern more clearly, factors associated with progression for this subset of high-risk patients. Also, follow-up was relatively short, limiting statements regarding long-term outcomes with this approach with further potential events possible with longer follow-up. However, these results add further validation to the concept of risk-stratified prospective BCRL surveillance for breast cancer patients at risk of developing BCRL. Additional long-term outcomes data with such an approach will further identify key cohorts of patients for prospective surveillance such that resource utilization is optimally cost-effective.

## Conclusion

The results of this analysis underscore previously published data on the efficacy of prospective BCRL surveillance and early intervention using BIS. Of the 93 high-risk patients prospectively followed and managed in this structured BCRL protocol, only 11% required CDP and only 3% required continued therapy. These excellent outcomes are superior to contemporary studies of conventional measures reporting BCRL rates in similarly treated high-risk patients.

## Ethics Statement

Collection of data and the analysis was given institutional review board (IRB) approval [WIRB Exemption Determination under 45 CFR 46.101(b)(4)].

## Author’s Note

Presented in part at the American Society of Clinical Oncology Annual Meeting, Chicago, Illinois, June 2017.

## Author Contributions

PW was involved in project conception, data gathering, data analysis, manuscript writing and approval. CS was involved in project conception, data analysis, manuscript writing and approval. FV was involved in project conception, data analysis, manuscript writing and approval. AC was involved in project conception, data collection, data analysis, manuscript writing and approval.

## Conflict of Interest Statement

PW—funding provided for data collection/analysis. CS—Scientific Consultant, ImpediMed. FV—CMO, ImpediMed. AC—No conflicts of interest. The reviewer KM and handling Editor declared their shared affiliation.
